# Supplementary material for: The Clinical Characteristics and Treatments for Large Cell Carcinoma Patients Older than 65 Years Old: A Population-Based Study
Source: Cancers (Basel). 2022 Oct 25;14(21):5231. doi: 10.3390/cancers14215231 (PMC9658669; doi:10.3390/cancers14215231)
Supplement: Supplementary file 1 [file cancers-14-05231-s001.zip › cancers-1835471-supplementary.pdf]

Table S1. Baseline characteristics of patients with LCC and other types of NSCLC after PSM

**Characteristics**	**LCC (n=11349)**	**Others (n=11349)**
**Race**		
White	9243(81.4%)	9312(82.1%)
Black	1568(13.8%)	1437(12.7%)
Asian and others	528(4.7%)	582(5.1%)
Unknown	10(0.1%)	18(0.2%)
**Sex**		
Male	6618(58.3%)	6720(59.2%)
Female	4731(41.7%)	4629(40.8%)
**Year of diagnosis**		
2004-2007	5487(48.3%)	4624(40.7%)
2008-2011	3508(30.9%)	3764(33.2%)
2012-2015	2354(20.7%)	2961(26.1%)
**Region**		
East	5979(52.7%)	5729(50.5%)
Northern Plains	1237(10.9%)	1346(11.9%)
Southwest	343(3.0%)	313(2.8%)
Alaska and Pacific Coast	3790(33.4%)	3961(34.9%)
**Tumor location**		
Upper lobe	5857(51.6%)	5729(50.5%)
Middle lobe	465(4.1%)	413(3.6%)
Lower lobe	2573(22.7%)	3147(27.7%)
NOS	1761(15.5%)	1260(11.1%)
Overlapping lesion	137(1.2%)	144(1.3%)
Main bronchus	546(4.8%)	612(5.4%)
Trachea	10(0.1%)	23(0.2%)
**Grade**		
Grade I	28(0.2%)	364(3.2%)
Grade II	95(0.8%)	1862(16.4%)
Grade III	3368(29.7%)	2731(24.1%)
Grade IV	2781(24.5%)	106(0.9%)
Unknown	5077(44.7%)	6286(55.4%)
**Stage**		
Stage I	1269(11.2%)	1501(13.2%)
Stage II	668(5.9%)	810(7.1%)
Stage III	2972(26.2%)	3572(31.5%)
Stage IV	5735(50.5%)	4423(39.0%)
Unknown	705(6.2%)	1043(9.2%)
**Laterality**		
Right-origin of primary	6282(55.4%)	6162(54.3%)

Left - origin of primary	4420(38.9%)	4698(41.4%)
Bilateral, single primary	172(1.5%)	142(1.3%)
Paired, but no laterality	401(3.5%)	268(2.4%)
Others	74(0.7%)	79(0.7%)
**Lymphadenectomy**		
0-3 LNRs	585(5.2%)	531(4.7%)
≥4 LNRs	1683(14.8%)	1883(16.6%)
Biopsy or aspiration	542(4.8%)	542(4.8%)
Sentinel biopsy	14(0.1%)	17(0.1%)
None	8216(72.4%)	8081(71.2%)
Unknown	309(2.7%)	295(2.6%)
**Surgery record**		
Yes	2419(21.3%)	2655(23.4%)
No	8854(78.0%)	8585(75.6%)
Unknown	76(0.7%)	109(1.0%)
**Radiation sequence**		
No radiation and/or surgery	9951(87.7%)	10076(88.8%)
Radiation after surgery	1218(10.7%)	1070(9.4%)
Radiation prior to surgery	156(1.4%)	163(1.4%)
Radiation before and after S	12(0.1%)	23(0.2%)
Intraoperative radiation	3(0.0%)	4(0.0%)
Sequence unknown, but both given	14(0.1%)	8(0.1%)
Surgery before and after radiation	0(0.0%)	3(0.0%)
Radiation in and before/after surgery	0(0.0%)	2(0.0%)
**Radiation record**		
Beam radiation	4680(41.2%)	4727(41.7%)
Beam with implants or isotopes	8(0.1%)	19(0.2%)
Implant or radioisotopes	18(0.2%)	19(0.2%)
Radiation, but not specified	58(0.5%)	79(0.7%)
No radiation	6445(56.8%)	6399(56.4%)
Unknown	140(1.2%)	106(0.9%)
**Chemotherapy record**		
Yes	5408(47.7%)	5293(46.6%)
No/unknown	5941(52.3%)	6056(53.4%)
**Tumor Size**		
≤1cm	8567(75.5%)	8667(76.4%)
>1, ≤2cm	7(0.1%)	4(0.0%)
>2, ≤3cm	9(0.1%)	12(0.1%)
>3, ≤4cm	9(0.1%)	6(0.1%)
>4 cm	6(0.1%)	3(0.0%)
Unknown	2751(24.2%)	2657(23.4%)
**Bone Metastasis**		
Yes	759(6.7%)	613(5.4%)
No	2937(25.9%)	3829(33.7%)

Unknown	7653(67.4%)	6907(60.9%)
**Brain Metastasis**		
Yes	668(5.9%)	390(3.4%)
No	3024(26.6%)	4034(35.5%)
Unknown	7657(67.5%)	6925(61.0%)
**Liver Metastasis**		
Yes	610(5.4%)	374(3.3%)
No	3086(27.2%)	4064(35.8%)
Unknown	7653(67.4%)	6911(60.9%)
**Lung Metastasis**		
Yes	551(4.9%)	557(4.9%)
No	3125(27.5%)	3896(34.3%)
Unknown	7673(67.6%)	6896(60.8%)
**First malignant primary indicator**		
Yes	9213(81.2%)	8807(77.6%)
No	2136(18.8%)	2542(22.4%)
**Age at diagnosis**		
<65	4300(37.9%)	3819(33.7%)
≥65	7049(62.1%)	7530(66.3%)
**Insurance status**		
Any Medicaid	993(8.7%)	1052(9.3%)
Insured or no specifics	5655(49.8%)	6368(56.1%)
Uninsured	260(2.3%)	240(2.1%)
Unknown	4441(39.1%)	3689(32.5%)
**Marital status**		
Married or domestic partner	5975(52.6%)	5922(52.2%)
Divorced/separated/single/widowed	4995(44.0%)	4993(44.0%)
Unknown	379(3.3%)	434(3.8%)
**High school education (%)**		
≤10	2050(18.1%)	2202(19.4%)
>10, ≤20	5984(52.7%)	5822(51.3%)
>20, ≤30	2981(26.3%)	2929(25.8%)
>30	332(2.9%)	395(3.5%)
Unknown	2(0.0%)	1(0.0%)
**Median family income (dollar, in tens)**		
≤5000	1682(14.8%)	1682(14.8%)
>5000, ≤7000	5745(50.6%)	5470(48.2%)
>7000, ≤9000	2854(25.1%)	2909(25.6%)
>9000	1066(9.4%)	1287(11.3%)
Unknown	2(0.0%)	1(0.0%)

Abbreviations: NSCLC: non-small cell lung cancer; LCC: large cell carcinoma.
